# Bio-Inspired Hydrogel–Elastomer Actuator with Bidirectional Bending and Dynamic Structural Color

**DOI:** 10.3390/molecules28196752

**Published:** 2023-09-22

**Authors:** Yongqing Xia, Yaru Meng, Ronghua Yu, Ziqi Teng, Jie Zhou, Shengjie Wang

**Affiliations:** Department of Biological and Bioenergy Chemical Engineering, College of Chemical Engineering, China University of Petroleum (East China), Qingdao 266580, China; z22030134@s.upc.edu.cn (Y.M.); z21030159@s.upc.edu.cn (R.Y.); z23030157@s.upc.edu.cn (Z.T.); z22030136@s.upc.edu.cn (J.Z.); sjwang@upc.edu.cn (S.W.)

**Keywords:** hydrogel actuator, structural color, thermoresponsive hydrogel/microgel, gripper

## Abstract

In nature, some creatures can change their body shapes and surface colors simultaneously to respond to the external environments, which greatly inspired researchers in the development of color-tunable soft actuators. In this work, we present a facile method to prepare a smart hydrogel actuator that can bend bidirectionally and change color simultaneously, just like an octopus. The actuator is fabricated by elastomer/hydrogel bilayer and the hydrogel layer was decorated with thermoresponsive microgels as the photonic crystal blocks. Compared with the previously reported poly(N-isopropylacrylamide) hydrogel-based bilayer hydrogel actuators, which are generally limited to one-directional deformation, the elastomer/hydrogel bilayer actuator prepared in our work exhibits unique bidirectional bending behavior in accordance with the change of structural color. The bending degrees can be changed from −360° to 270° in response to solution temperatures ranging from 20 °C to 60 °C. At the same time, the surface color changes from red to green, and then to blue, covering the full visible light spectrum. The bending direction and degree of the hydrogel actuator can easily be adjusted by tuning the layer thickness ratio of the elastomer/hydrogel or the composition of the hydrogel. The color-tunable hydrogel-elastomer actuator reported in this work can achieve both programmable deformations and color-changing highly resembling the natural actuating behaviors of creatures.

## 1. Introduction

In nature, some creatures can change their body shapes and surface colors simultaneously to respond to external environments. Cuttlefish, octopus, and squid are typical examples that have the versatile capability to use body shapes and coloration to avoid predators. This unique ability has greatly inspired researchers working to develop color-tunable soft actuators [1,2,3,4,5,6].

Hydrogel actuators, which are soft and wet, similar to living organisms, can respond to various external stimuli, such as heat [7,8,9,10], pH [11,12,13], light [14,15,16,17,18,19], and chemicals [20,21,22] to achieve controllable and reversible shape formation. Therefore, they have shown promising applications including use in soft robots, artificial muscles, valves, and so on. Though various approaches have been reported to release complex deformations/movements of hydrogel actuators, the fabrication of anisotropic structures, especially anisotropic bilayers, has become more and more popular. Commonly, the bilayers are composed of an active layer and a passive layer, the bending of the hydrogel can be achieved by the response of the active layer to external stimuli. Poly(N-isopropylacrylamide) (PNIPAM) hydrogel is well known for its thermoresponsivity, with a low volume phase transition temperature (VPTT) of ~32 °C. Thus, PNIPAM hydrogel is the most appealing active layer for the fabrication of temperature-responsive hydrogel actuators. Unfortunately, the bending direction of PNIPAM-based hydrogel actuators is commonly limited by the passive layer, resulting in the actuator bending to one-directional deformation or within a small range of angles, which limits the application of the actuators. For example, Cheng et al. prepared a bilayer hydrogel soft actuator that contained poly(N-isopropylacrylamide) and poly(2-(dimethylamino) ethyl methacrylate), respectively. The hydrogel actuator showed reversible “line to coil” bending behavior under temperature variation (15–45 °C), due to inhomogeneous shrinkage of different layers [23]. Zhang et al. reported a bilayer structural color hydrogel actuator employing a hybrid inverse opal scaffold to join the poly(acrylic acid-co-acrylamide) layer and the poly(N-isopropylacrylamide) layer together. The bending ability of the bilayer structural color hydrogels under thermo-stimulation between 0 °C and 50 °C had the largest range of bending angles, from −30° to 360° [24]. To endow PNIPAM hydrogel actuators with bidirectional bending behavior, hydrogel layers with upper critical solution temperature (UCST), such as poly(N-acryloyl glycinamide), poly(acrylic acid), poly(3-(1-(4-vinylbenzyl)-1H-imidazol-3-ium-3-yl) propane-1-sulfonate) were incorporated with the PNIPAM hydrogel layer [25,26,27]. However, the mechanical strength of the hydrogels is insufficient, limiting their application range. PDMS (poly-dimethylsiloxane) is a polymeric silicone material with unique optical properties and biocompatibility. It is striking that PDMS has very good mechanical properties, which makes it a potential active material for tunable devices [28,29,30,31].

In addition to shape change, cephalopods control body color for communication and camouflage; this inspired researchers to pursue soft actuators with tunable color. As well as chemical colors incorporated in hydrogel actuators, physical colors generated by the selective interaction of light with periodic micro/nanostructures have recently attracted broad attention. Since structural colors can be synchronously expressed with the deformation of the hydrogel actuators, the actuation status and changes of the hydrogel actuators can therefore be monitored and reported by the structural colors in real time.

Herein, we propose a new strategy to design and synthesize a bidirectional bending hydrogel actuator with tunable structural color beyond the limitation of observing angles. The hydrogel actuator is constructed using a PNIPAM hydrogel layer with an elastomer polydimethylsiloxane (PDMS) layer. The higher tensile strength of the PDMS layer increases the overall stiffness of the hydrogel actuator. Different from PNIPAM hydrogels with inversed opal structure, thermoresponsive microgels which can tune their size were used as the building blocks, decorated onto the PNIPAM layer to produce the structural color; the color is low angle-dependent due to the amorphous state of the microgels. Therefore, the hydrogel actuator can generate color signals due to the bending, and the actuating process can be monitored by the color in real-time. The bidirectionally bending soft actuator with tunable structural color may have potential applications in intelligent robots, smart grippers, etc.

## 2. Results and Discussion

### 2.1. Fabrication of Bilayer Anisotropic PNIPAM-PDMS Actuator

**Figure 1:**
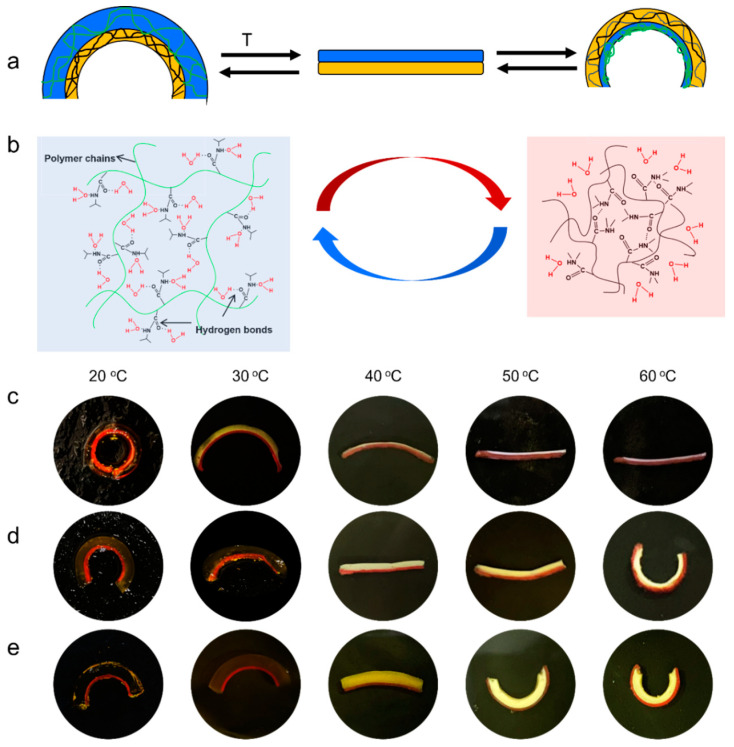
(**a**) The schematic illustration for bending behavior of the PNIPAM/PDMS bilayer hydrogel stripes in response to temperature. (**b**) The schematic illustration for the swelling and shrinkage states of the active PNIPAM hydrogel layer. Photographs of the synergistic bilayer stripes made of PNIPAM/PDMS, thickness ratio of (**c**) 1:1; (**d**) 3:1, and (**e**) 5:1.

Soft materials have recently risen in popularity for a wide variety of engineering applications, particularly in the areas of soft actuators. However, most soft actuators are designed with hydrogel, and mechanical weakness has limited their applications. Owing to its biological compatibility, gas permeability, and mechanical strength, polydimethylsiloxane (PDMS) is considered a promising passive layer for soft actuators to increase the mechanical strength of actuators. In this work, PNIPAM hydrogel and PDMS were combined via surface grafting of monomer-containing hydrogels onto a benzophenone-treated PDMS surface (Figure S1). Thus, the bending of the PNIPAM/PDMS bilayer actuator was controlled by the coefficient of thermal expansion difference between the PNIPAM and PDMS layer (Figure 1a). At temperatures below VPTT, the PNIPAM hydrogel layer swells and the weight of the PNIPAM hydrogel layer is greater than that of the PDMS layer, and the bilayer bends to the PDMS layer. However, when the temperature is above the VPTT, the PNIPAM hydrogel layer shrinks while the PDMS layer remains constant, resulting in the bending of the bilayer film to the PNIPAM layer (Figure 1b). The PNIPAM/PDMS bilayer actuator exhibits an advantage in bending amplitude over the conventional single-active-layer bilayer hydrogel actuators, which usually display one-directional bending.

The bending amplitude of the PNIPAM/PDMS bilayer actuator can be controlled by various factors, such as the crosslinking degree of the PNIPAM hydrogel layer, the thickness ratio of the PNIPAM/PDMS, and the length of the actuator strip. To demonstrate this, PNIPAM hydrogels with different crosslinking degrees (13%, 16%, 20%, and 26%) and different PNIPAM/PDMS thickness ratios (1:1, 3:1, and 5:1) were prepared to make strips. Among these comparison groups, the cross-linking degree had no obvious influence on the bending degree (Figure S2); thus, the PNIPAM hydrogel with a 20% crosslinking degree was used in the subsequent experiments. However, the thickness ratio plays a key role in bending behavior. When the thickness ratio of the PNIPAM/PDMS was 1:1, the bilayer strip bent towards the PDMS layer at 25 °C, and toward a flat strip at 50 °C, without further bending towards the PNIPAM layer at 60 °C (Figure 1c). Interestingly, the bilayer strips with thickness ratios of 3:1 and 5:1, respectively, showed better actuating performance, both bending towards the PDMS at low temperatures and towards the PNIPAM layer at higher temperatures. The thickness ratio of 3:1 showed a larger bending angle to the PDMS layer at low temperature, while the thickness ratio of 5:1 had a larger bending angle to the PNIPAM layer at high temperature (Figure 1d,e). At low temperature (20 °C), the thinner the PNIPAM layer, the larger was the bending angle towards the PDMS layer, from −360° (1:1) to −263° (3:1), and then to −180° (5:1). However, at high temperature (60 °C), the positive bending towards the PNIPAM exhibited an opposite trend. The thicker the PNIPAM layer, the larger was the bending angle towards the PNIPAM layer, from 0° (1:1) to 247° (3:1), and then to 265° (5:1).

**Figure 2:**
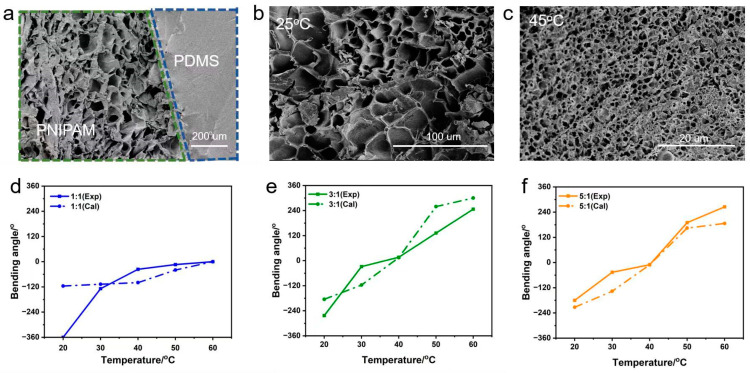
Cross-section SEM images of lyophilized bilayer hydrogel. (**a**) The bonding interface between the PDMS layer and the PNIPAM hydrogel layer. (**b**) The PNIPAM hydrogel layer at 25 °C. (**c**) The PNIPAM hydrogel layer at 45 °C. The plots of the bending angle were obtained from experimental and theoretical calculations of the PNIPAM/PDMS stripes with thickness ratios of (**d**) 1:1, (**e**) 3:1, and (**f**) 5:1 at different temperatures.

The cross-sectional SEM image of the hydrogel–PDMS film shows clearly that the porous hydrogel and the nonporous elastomer were connected (Figure 2a). It should be noted that the pore sizes of the PNIPAM hydrogel changed with temperature. At 25 °C (below the VPTT), the pore sizes were much larger than at 45 °C (above the VPTT) (Figure 2b,c), which means the PNIPAM hydrogel layer transferred from hydrophilic to hydrophobic, the hydrogel shrunk, and water was squeezed from the hydrogel. PNIPAM hydrogels are known to have poor mechanical strength, which hinders their practical application. The storage modulus was about only 1 KPa at 25 °C, and a minor modulus switching occurred between 25 °C and 40 °C. As the temperature increased from 40 °C to 60 °C, the storage modulus increased clearly and was about 5.5 KPa at 60 °C (Figure S3a). The reason can be attributed to the hydrophobicity of PNIPAM hydrogels at temperatures above VPTT. However, the stress of the PDMS film can reach 260 KPa, and the strain can increase to 120% (Figure S3b). This means that the PDMS film can provide good mechanical support for PNIPAM hydrogel.

To calculate the bending angles in theory, modified Timoshenko bimetallic thermostats theory was used to calculate the bending angles, in which the relationship between the thickness ratio and curvature of bilayers consisting of distinct materials can be predicted as follows [32]:

$$\alpha =\frac{L_{0}}{t_{1}}\frac{6\eta \left(1+\eta \right)\lambda_{1}\lambda_{2}\left(\lambda_{1}-\lambda_{2}\right)\xi }{\eta^{4}\lambda_{1}^{2}\xi^{2}+\left(4\eta +6\eta^{2}+4\eta^{3}\right)\lambda_{1}\lambda_{2}\xi +\lambda_{2}^{2}}$$

where α is the radian of the bilayer, and t_1_ and t_2_ are the thickness of the PNIPAM and PDMS layers, respectively. L_0_ represents the initial length of the bilayer actuator (for the PNIPAM and PDMS layers), η = t_2_\t_1_, ξ = E_2_\E_1_, and λ_1_ and λ_2_ are the deformation ratios of the PNIPAM and PDMS layers, which can be obtained from the change ratio of the length (L/L_0_). At different temperatures, the length of the PNIPAM layer expands or shrinks, while the PDMS layer remains unchanged. The results showed that the experimental values fit well with the theoretical values, especially for bilayers with a PNIPAM/PDMS thickness ratio of 5:1 (Figure 2d–f). The largest deviation between the experimental and theoretical values occurred at 20 °C with a thickness ratio of 1:1. The reason is probably due to the small thickness of the PNIPAM actuators, which are not well accounted for in the theoretical models.

**Figure 3:**
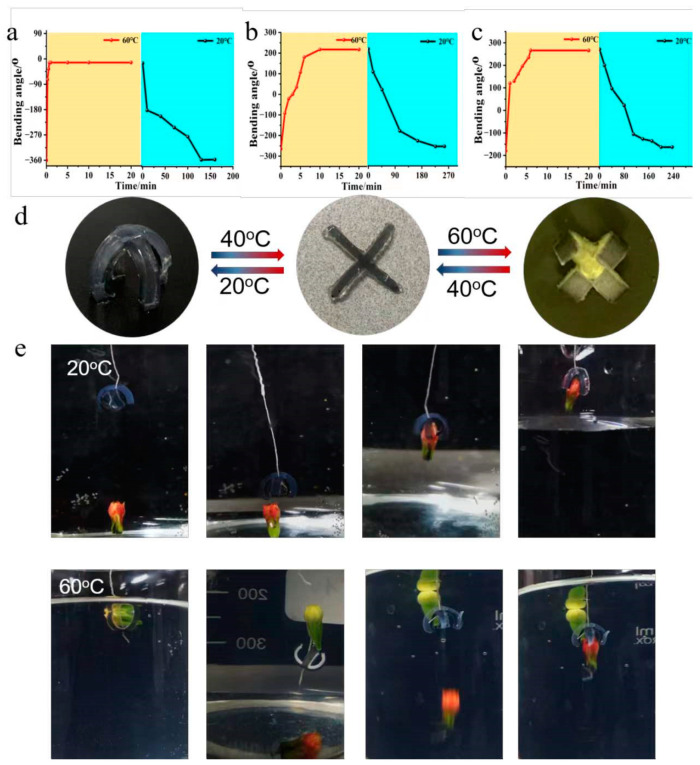
Bending kinetics of the PNIPAM/PDMS bilayer hydrogel actuators with different thickness ratios: (**a**) 1:1, (**b**) 3:1, and (**c**) 5:1 in response to temperature (20–60 °C). (**d**) Bending behavior of PNIPAM/PDMS four-arm gripper with 5:1 ratio, actuated by temperature (20 °C–40 °C–60 °C). (**e**) An entire actuation procedure for the gripper (circled in white) to capture, transport, release, and reverse capture two different objects (red and yellow) in response to temperature change.

To further quantify the bending kinetics of bilayer hydrogels in response to the changes from cool water to hot water, the stripes with 20% cross-linking degree and thickness ratios of 1:1, 3:1, and 5:1 were used to study their response times (Figure 3a–c). Results showed that at 20 °C, all strips showed a negative bending when put into 60 °C hot water; they rapidly changed their initial bending from −360°, −270°, and −180° to 0° within ~1 min (the first stage), then continuously and gradually increased their bending to a positive curvature (0–270°) in ~5 min (the second stage). The response time of the hydrogel–elastomer actuator (5:1) to complete the bending behavior at 60 °C is detailed in Figure S4. However, the recovery speeds of the strips were slower, all needing over 3 h, exhibiting slow reswelling behavior typical of PNIPAM hydrogel [33,34,35]. The bending angle of the PNIPAM/PDMS bilayer film remained constant over 10 actuation cycles, demonstrating stable bending–recovery performance and good reversibility (Figure S5). As a result, a cross-shaped actuator with a PNIPAM/PDMS thickness ratio of 5:1 was further fabricated, because it had the largest range of bending angles, and its bidirectional bending behavior can be used as an intelligent gripper to grip objects reversely (Figure 3d). When put into cold water, the gripper gradually bent its arms downward to grasp the red object, and held it tightly for further transport back (Figure 3e). When the water temperature increased, the gripper arms released the red object and then bent to the PNIPAM layer to grab the yellow object reversely, completing the grab–release–reverse grab function. When the temperature decreased, the yellow object was released, and the red object was grabbed again. This process is reversible and different from previously reported smart hydrogel grippers.

### 2.2. Color-Tunable Hydrogel Actuator

The dynamic color and shape change of the cephalopod inspired the fabrication of a color-tunable hydrogel actuator. SEM and TEM proved that the skin of the cephalopod is composed of photonic crystals. According to the Bragg diffraction equation, the position of the reflection peak of the photonic crystal is affected by the incident angle, effective refractive index, and lattice spacing. Therefore, these parameters can be changed by external stimuli to realize the control of the reflection peak position of the photonic crystal. 

**Figure 4:**
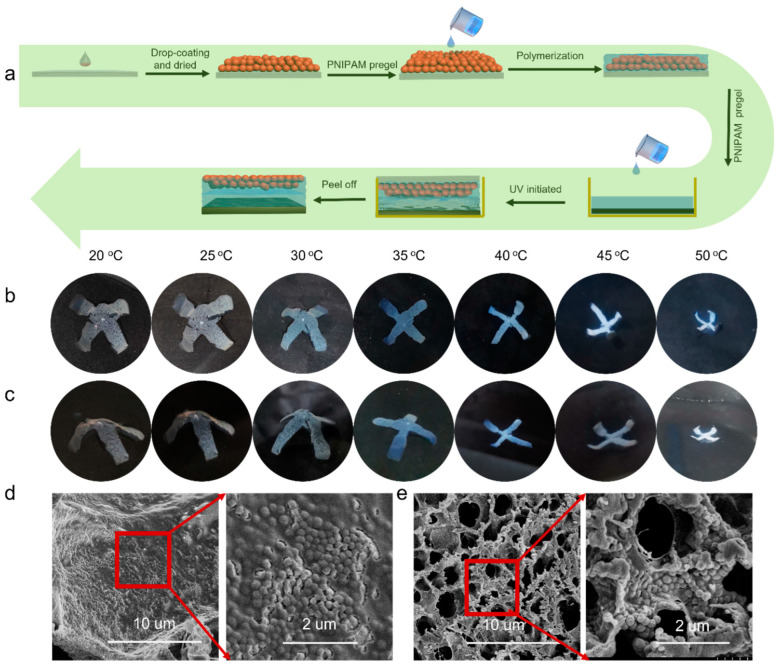
(**a**) The preparation process of the structural-colored PNIPAM/PDMS actuator. (**b**) The obtained actuator showed both shape deformation and color change (observing from the top). (**c**) Photographs taken from the side. (**d**) SEM images of the structural colored surface at 25 °C and the areas in red boxes enlarged. (**e**) SEM images of the structural colored surface at 45 °C and the areas in red boxes enlarged.

In our previous work [36], PNIPAM hydrogel embedded with the close-contact PNIPAM microgel film displayed tunable structural color under temperature stimuli. Since the interaction between the PDMS and PNIPAM hydrogel must be achieved under UV irradiation, a long reaction time would disturb the close-contact state of the microgels and lose the structural color. We tried to embed the PNIPAM microgels in the medium layer between the PNIPAM and PDMS layers. Results showed that the formed hand-shape bilayer had excellent bending behavior without obvious color changing (Figure S6). To integrate the microgel film onto the outer surface of the PNIPAM hydrogel, a patching method was proposed to prepare the structural colored hydrogel–elastomer actuator. The preparation procedure is demonstrated in Figure 4a. An optical hydrogel patch using PNIPAM microgels as the building blocks was first prepared by casting PNIPAM pregel solution onto the dried microgel film to form the patch using the APS-TEMED initiating system, then the patch was integrated with the PDMS layer using the NIPAM pregel solution as the adhesive medium; this procedure was achieved by UV initiation. Figure 4b demonstrates the successful preparation of the structural colored PNIPAM/PDMS actuator. The obtained bilayer showed a pale red color at room temperature and was bent to the PDMS layer. As the temperature increased, the color of the hydrogel actuator gradually blue-shifted, along with bending toward the PDMS layer. During this process, with the temperature increase, the spacing distance (microgels’ diameter) decreased because of the shrinking, and according to Bragg’s equation, this would lead to the decreasing of reflection wavelength λ. More importantly, the structural color of the actuator showed no obvious difference when observed from different angles (Figure 4c). SEM images showed the structured colored surface had large pores, which were like those of the common PNIPAM hydrogel. However, the enlarged area indicates that the PNIPAM surface was covered by dense microgels (Figure 4d), which presented a disordered arrangement. At 50 °C, the morphology of the colored surface was like the PNIPAM hydrogel at temperatures above VPTT, with more dense pores than that at 20 °C (Figure 4e). The disordered structure was responsible for the angle-independent color [37,38,39,40,41]. This result was quite different from previous work in which an inversed optical PNIPAM hydrogel layer was used to reflect color. In Zhao’s work, with the temperature increasing, the structural color turned from green to red, and the reflective wavenumber red-shifted.

**Figure 5:**
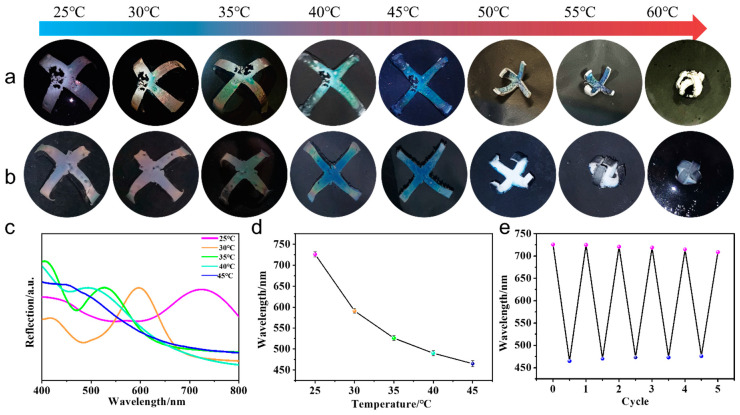
(**a**) Color change and shape deformation process of the PNIPAM/PDMS actuator with MWCNTs added in the PNIPAM layer. (**b**) Color change and shape deformation process of the PNIPAM/PDMS actuator with MWCNTs added in the PDMS layer. (**c**) The reflection spectra of the colored surface of (**a**,**d**) the corresponding reflection peaks. (**e**) The reversible cycle of the reflection peaks of the actuator between 25 °C and 45 °C.

Because the microgels were amorphous in this system, the major part of the incident light was able to transmit the transparent hydrogel and PDMS layers and reach the background, and the reflected light from the back substrate lowered the reflection intensity of the microgel film, resulting in unvivid structural color. In previous reports, small black substances, such as carbon black, cuttlefish ink, and polydopamine (PDA) nanoparticles have been added to the amorphous colloidal photonic crystals to enhance structural colors [37,39,42]. However, the addition of black materials into the microgel film may decrease the total amount of reflection. In this context, the black materials MWCNTs were added to the PNIPAM hydrogel layer and the PMDS layer, respectively. Results showed the color visibility of the actuator was somewhat brighter than these images taken without MWCNTs, the structural colors with PNIPAM/MWCNTs were more vivid than those with PDMS/MWCNTs, and the exhibited colors were different at temperatures above 50 °C. When the temperature increased from 25 °C to 60 °C, the structural color of the actuator with MWCTNs in the PNIPAM layer turned from its original violet to golden red at 30 °C, golden green at 35 °C, green at 40 °C, and blue at a temperature from 45 °C to 60 °C (Figure 5a). The reflection spectra and reflection peaks proved the blue shifting of the structural color with increasing temperature (Figure 5c,d). However, for the actuator with MWCNTs in the PDMS layer, the color disappeared at temperatures above 50 °C (Figure 5b). The reason may be due to the medium PNIPAM layer between the structural colored film and PDMS layer becoming white at temperatures above 50 °C and able to scatter light strongly, which caused the structural color to be invisible on the white background. Furthermore, their reflection spectra also showed a difference. For the PDMS/MWCNTs actuator, the reflection spectra showed a single peak curve at each temperature (Figure S7), while for the PNIPAM/MWCNTs actuator, second peaks appeared at a short wavelength (around 400 nm), which may have been produced by the disordered arrangement of the microgels (Figure 5c). It should be noted that the shapes of both actuators changed from the original vertical bending towards the PDMS layer to flat at 40 °C, then bent in reverse towards the hydrogel layer, eventually closing at 60 °C. The change of the reflection peaks was reversible (Figure 5e).

## 3. Experimental Section

### 3.1. Materials

The Sylgard 184 silicon elastomer kit was purchased from Dow Corning (Midland, MI, USA). N-isopropylacrylamide (NIPAM), styrene (ST), acrylamide (AM), acrylic acid (AA), N, N’-methylene bis (Acrylamide)(MBA), poly(ethylene glycol) diacrylate (PEGDA, Mw 700) and 2-Hydroxy-40-(2-hydroxyethoxy)-2-methylpropiophenone (Irgacure 2959) were purchased from Sigma-Aldrich (St. Louis, MO, USA) and used without further purification. Ammonium persulfate (APS) was purified from water by recrystallization. N, N, N’, N’ -tetramethylene ethylenediamine (TEMED) was purchased from China Pharmaceutical Co., Ltd. (Beijing, China) and used as received. Carboxyl multiwalled carbon nanotubes (MWCNTs) were bought from Nanjing XF NANO Materials Tech Co., Ltd. (Nanjing, China). A Milli-Q ^®^ Advantage A10 water purification system (Millipore, Bedford, MA, USA) was utilized to generate pure water (resistivity higher than 18 MΩ cm). 

### 3.2. Preparation of the Thermoresponsive PNIPAMST Microgels

The thermoresponsive poly(N-isopropylacrylamide-co-styrene) (PNIPAMST) microgel emulsion was prepared by surfactant-free precipitation polymerization, as in our previous work [36]. Typically, 1.500 g of NIPAAm, 0.500 g of styrene, and 0.041 g of MBA were added in 190 mL of water, and the reaction mixture was transferred to a four-necked, round-bottom flask equipped with a condenser and nitrogen inlet and then heated to 70 °C under the protection of nitrogen. After 1 h, initiator solution (0.120 g of APS dissolved in 10 mL of water) was dropped into the flask to initiate polymerization. The reaction was continued for 4 h while keeping the reaction in a nitrogen environment, then cooled down to room temperature under stirring. The obtained microgel emulsion was used directly without further purification. The hydrodiameters of the microgels were characterized by dynamic light scattering (DLS, Zetasizer Nano instrument from Malvern Instruments Ltd. (Malvern, UK), with the detector positioned at a scattering angle of 173°) in the temperature range of 20–50 °C.

### 3.3. The Preparation and Modification of PDMS Film

PDMS substrates were prepared by mixing PDMS precursor with a curing agent in a 10:1 ratio by weight. After degassing, the mixture was evenly spread on a glass plate and cured at 90 °C for 2 h. The cured PDMS film was cut into pieces as samples for surface grafting.

The surface of the PDMS film was thoroughly cleaned with ethanol and deionized water, and then completely dried with nitrogen gas. Drops of benzophenone solution (10 wt% in ethanol) were added onto the PDMS to cover the entire surface for 2 min at room temperature, then the PDMS was washed with ethanol three times and completely dried with nitrogen gas.

### 3.4. Preparation of the Hydrogel-Elastomer Actuator with Structural Color

Preparation of PNIPAM pregel solution involved mixing 8 mL of a carefully degassed aqueous pre-gel solution (17 wt% NIPAM, 10 wt% Am, 13–25 wt% PEGDA and 0.2 wt% Irgacure 2959). The mixture was mixed quickly, and purged with nitrogen gas to remove any dissolved oxygen.

For preparation of microgel–hydrogel thin film, first, 200 μL of PNIPAMST microgel dispersion (1.5 wt%) was paint-coated on the glass coverslip (40 mm × 40 mm) and left to dry at 60 °C for 2 h to obtain a dry microgel film. Then, drops of PNIPAM pregel solution were added to the PNIPAMST microgel film, and irradiated with UV light at 20 °C for 30 min, so that the Irgacure 2959 initiated photopolymerization of the NIPAAm on the PNIPAMST microgel film surface.

For preparation of the hydrogel–elastomer actuator with structural color, the glass coverslip with polymerized PNIPAM hydrogel and microgel thin film was put upside down onto the PDMS film at determined space thickness, and the PNIPAM pregel solution was injected into the space between the PDMS and the glass coverslip. Then, polymerization of the sandwiched PNIPAM solution was initiated under UV for 1 h at 20 °C. Then, the obtained film was immersed in water to remove the glass coverslip.

### 3.5. Characterization of the Actuator

The hydrogel was equilibrated in water at 25 °C and 45 °C, respectively, and then quickly frozen in liquid nitrogen and further freeze-dried in a freeze drier for 2 days until all ice was sublimed. The surface and cross-section of the hydrogel were coated with a thin Au layer for scanning electron microscopy (SEM) imaging. The reflection spectra of the structural color of the hydrogel actuator at different temperatures were obtained by a fiber-optic spectrometer (USB2000+, Ocean Optics, Orlando, FL, USA). All the optical images of the hydrogels were captured with a mobile phone (Xiaomi Note) purchased from Xiaomi Technology Co., LTD. (Beijing, China).

## 4. Conclusions

Here we report a robust bidirectional bending hydrogel/elastomer optical actuation system, which was prepared by the interfacial bonding of a structural colored PNIPAM hydrogel layer and hydrophobic PDMS film with PNIPAM hydrogel as the medium. PNIPAM microgels were embedded in the outer layer of the PNIPAM and used as blocks for the photonic crystals; the synergistic effect between the thermoresponsive microgels and hydrogel endowed the hydrogel with blue-shifted structural color as temperature increased. The shrinkage of the PNIPAM hydrogel also made the bending direction change inversely, accompanying a temperature increase. Based on this process, dynamic structural color with bidirectional bending behavior of the actuator can be achieved. Furthermore, benefitting from the amorphous state of the microgels, the exhibited color was angle-independent. The reversible and bidirectional bending behaviors of soft actuators combined with tunable structural color expand their application scope.

## Data Availability

Data will be made available on request.

## Supplementary Materials

The following supporting information can be downloaded at: https://www.mdpi.com/article/10.3390/molecules28196752/s1. Figure S1: The scheme illustration of the formation process of the PNIPAM/PDMS bilayer hydrogel; Figure S2: The effect of crosslinking degree of PNIPAM hydrogel on the bending behavior of the bilayer actuator; Figure S3: The mechanical strength of the PDMS film and PNIPAM hydrogel; Figure S4: The response time of the hydrogel-elastomer actuator to complete the bending behavior at 60 °C; Figure S5: Reversible bending behavior of PNIPAM/PDMS bilayer hydrogel (5:1) switching between 20 °C and 60 °C in water; Figure S6: The shape deformation and color-changing of the hand-shaped PNIPAM/PDMS bilayer with PNIPAM microgels embedded between the layers under temperature stimuli; Figure S7: The reflection spectra of the colored surface of the actuator with MWCNTs embedded in the PDMS layer.
