# Supplementary material for: Bio-Inspired Hydrogel–Elastomer Actuator with Bidirectional Bending and Dynamic Structural Color
Source: Molecules. 2023 Sep 22;28(19):6752. doi: 10.3390/molecules28196752 (PMC10574087; doi:10.3390/molecules28196752)
Supplement: Supplementary file 1 [file molecules-28-06752-s001.zip › molecules-2602800-supplementary.pdf]

Supporting Information

1.

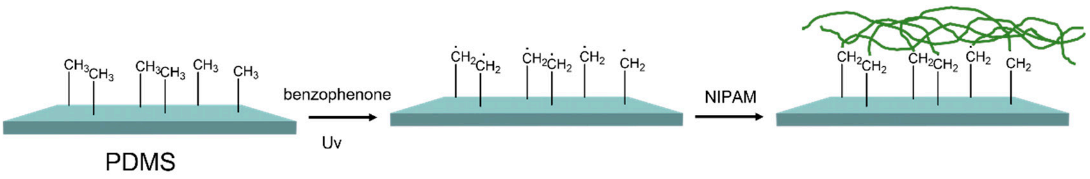

Figure S1. The scheme illustration of the formation process of the PNIPAM/PDMS bilayer hydrogel.

2.

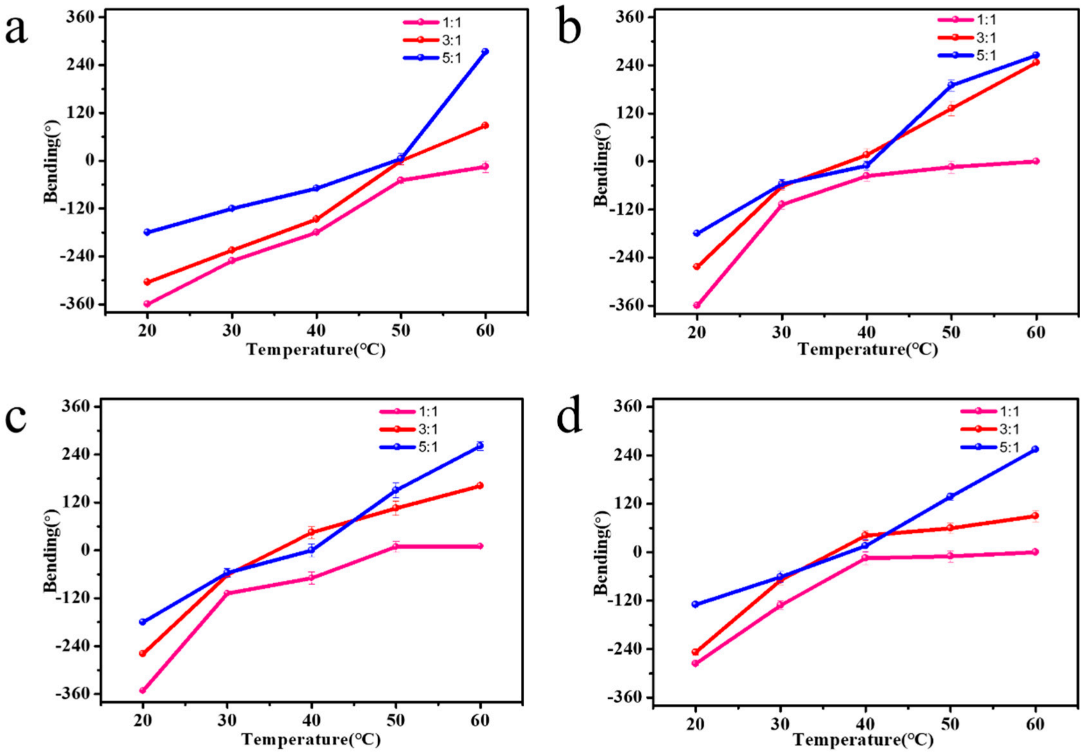

Figure S2. The effect of crosslinking degree of PNIPAM hydrogel (a) 13%, (b) 16%, (c) 20% and (d) 26% on the bending behavior of the bilayer actuator.

3.

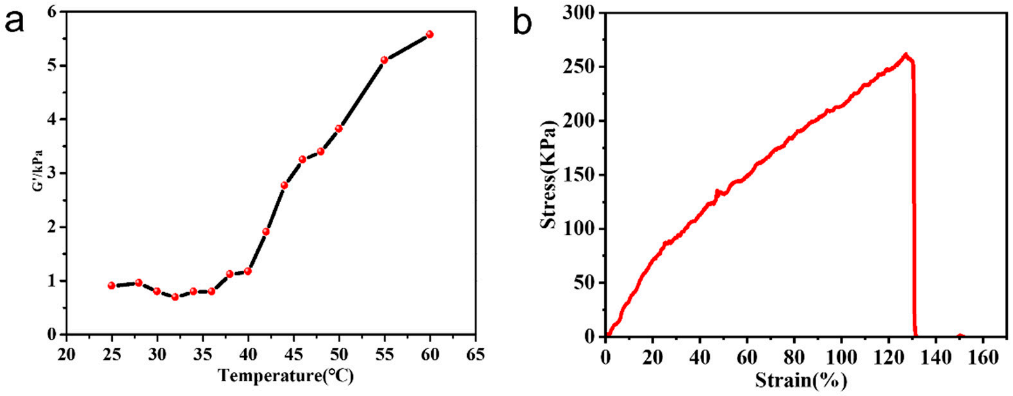

Figure S3. The mechanical strength of the PDMS film (a) and PNIPAM hydrogel (b). The mechanical strength of the latter was too weak to be determined by tensile.

4.

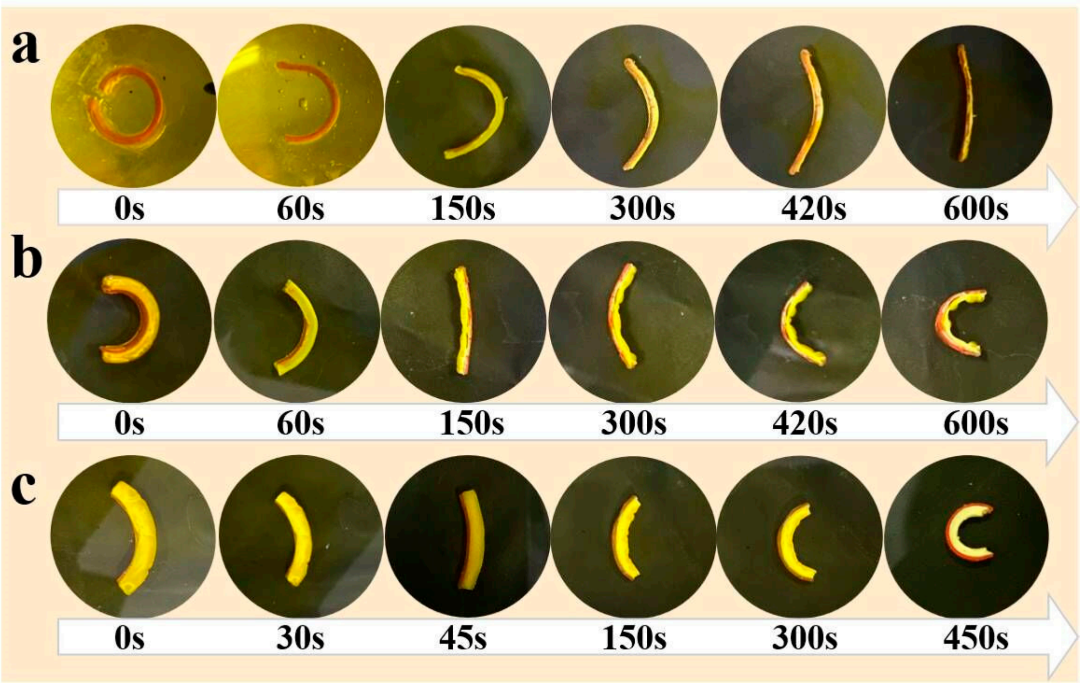

Figure S4. The response time of the hydrogel-elastomer actuator to complete the bending behavior at 60 °C. The thickness ratio of PNIPAM:PDMS was (a) 1:1, (b) 3:1 and (c) 5:1.

5.

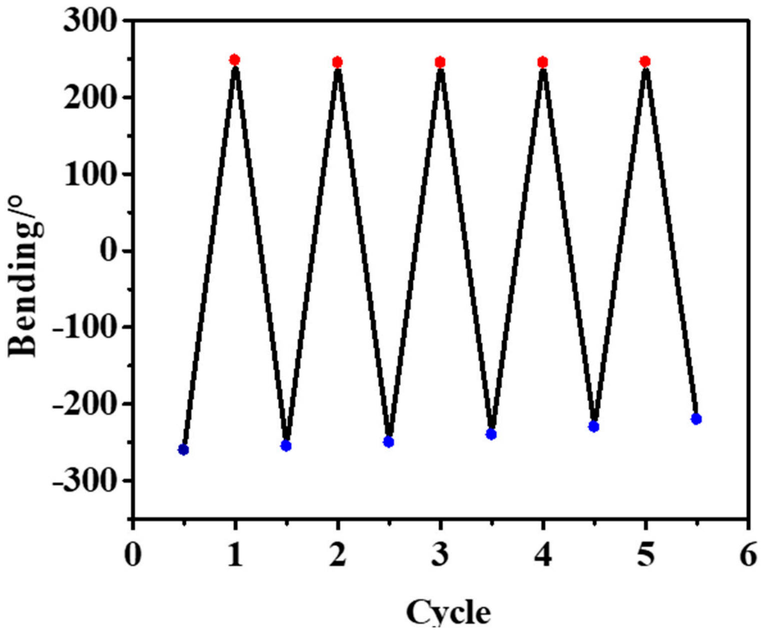

Figure S5. Reversible bending behavior of PNIPAM/PDMS bilayer hydrogel (5:1) switching between 20 °C and 60 °C in water.

6.

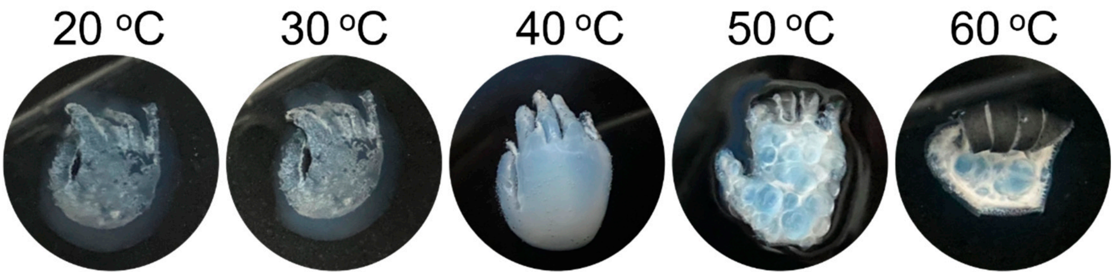

Figure S6. The shape deformation and color-changing of the hand-shaped PNIPAM/PDMS bilayer with PNIPAM microgels embedded between the layers under temperature stimuli.

7.

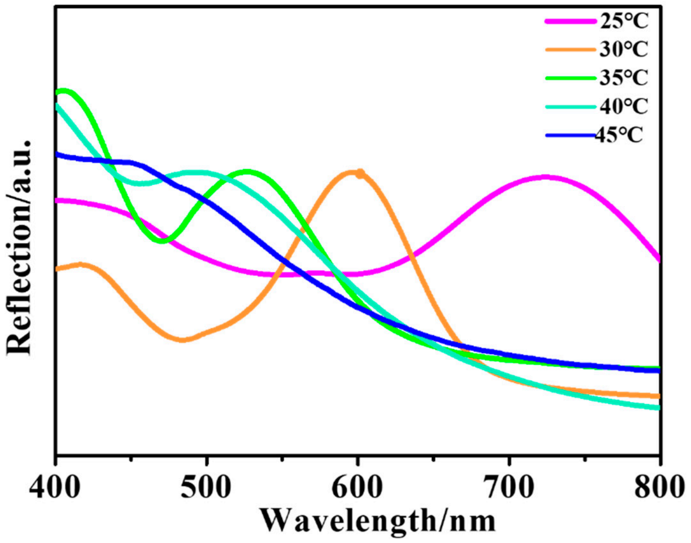

Figure S7. The reflection spectra of the colored surface of the actuator with MWCNTs embedded in the PDMS layer.
